# Influence of coping with stressful situations on changes in aerobic capacity and post-workout restitution coefficient in the period of immediate preparation for the European men’s cadet wrestling championship

**DOI:** 10.3389/fpsyg.2024.1433772

**Published:** 2024-07-26

**Authors:** Pola Jalowska, Marek Sokołowski, Adam Prokopczyk

**Affiliations:** Department of Sport and Defence Education, Poznan University of Physical Education, Poznań, Poland

**Keywords:** combat sport, wrestling, psychological preparation, stress, aerobic capacity

## Abstract

**Aim of the study:**

The research goal of the study was to determine the relationship between coping with stressful situations and the level of aerobic capacity and post-workout restitution, as well as the changes that occur between these variables through the period of training camp preceding international men’s championship competitions in age cadet. Two research hypotheses were verified. The athletes will maintain or improve the results obtained in the performance test and the post-workout restitution coefficient during the immediate preparation period for the European Championships (H1), and the style of coping with stressful situations significantly affects changes in aerobic capacity and the post-workout restitution coefficient during the immediate preparation period for the European Championships (H2).

**Materials and methods:**

The athletes of the National Men’s Team of Poland in classical style wrestling (*n* = 16). Coping with stressful situations was examined using the Coping Inventory for Stressful Situations (CISS). Aerobic capacity was analyzed using the Maximal Multistage 20-m Shuttle Run Test. The level of post-exercise restitution was calculated using the Klonowicz coefficient of restitution.

**Results:**

There was a significant increase in aerobic capacity levels (*p* < 0.001), a decrease in resting HR (*p* < 0.002), HR 1′ after the test (*p* < 0.0031), and HR 5′ after the test (*p* < 0.007). There was a significant correlation between emotional coping style and avoidant style focused on looking for social contacts vs. HR 3′ after the test and (*r* = 0.60; *p* < 0.015) and HR 5′ after the test (*r* = 0.57; *p* < 0.020). In addition, a correlation was noted between avoidant style and maximum aerobic speed (*r* = −0.64; *p* < 0.008), and avoidant style focused on substitute activities vs. distance and maximum aerobic speed (*r* = −0.72; *p* < 0.002).

**Conclusion:**

It is reasonable to implement psychological training and regular monitoring of mental preparation in the national men’s team training program for athletes competing in wrestling.

## 1 Introduction

In order to adequately prepare an athlete to compete in international wrestling championship competitions, a proper psychological and physiological background is vital (Sabirov, 2022; Korobeynikov et al., 2022a). For an athlete to achieve sporting success, it is necessary to have the ability to respond appropriately to stressful stimuli and to recover quickly from them, regardless of the age group in which they compete (Piepiora et al., 2021a; Atiya et al., 2022). Despite the fact that during a wrestling bout, the work in the anaerobic energy zone dominates (Marković et al., 2018; Pryimakov et al., 2020a), a properly arranged training process for an adolescent wrestler must also include aerobic capacity training as an integral training component (Demirkan, 2015). On a single day, a given athlete may fight several bouts and must be prepared to fight the next day in the final block or for the repechage bouts (and the bronze medal bout if the repechage is won). The volume of a single bout in classical style wrestling in the cadet (U17) age group is 2 rounds of 2 min each, with a 30-s break between rounds. In addition, from the junior (U20) age group onward, athletes wrestle longer—2 rounds of 3 min each with a 30-s break between rounds (United World Wrestling, 2023). For an athlete to succeed at international championship wrestling competitions, in addition to proper mental preparation, they must have an adequate level of aerobic capacity and post-exercise restitution. Such preparation will give you the ability to win a bout at a given championship and prepare for the next one as quickly as possible. In previous studies, the psychological characteristics of wrestlers, athletes participating in combat sports, individual sports, and team sports at the competitive level have been carried out (Tomczak et al., 2013; Piepiora, 2019, 2021; Piepiora and Petecka, 2020; Piepiora and Witkowski, 2020a,b; Piepiora and Piepiora, 2021; Piepiora et al., 2022; Piepiora and Naczyńska, 2023). Physical fitness, body composition, developmental age, aerobic capacity and its changes after a 3-month training period, eating habits, and somatic development were analyzed in young wrestlers (Clarke et al., 2013; Piepiora et al., 2017, 2018; Witkowski et al., 2018). Physiological criteria for the functional fitness of national team wrestling athletes have been determined (Pryimakov et al., 2020b). It has been shown that the proper aerobic-anaerobic preparation of wrestlers is one of the determinants of the rate of recovery after high-intensity training (Sawczyn et al., 2015). In addition, it has been shown that the level of aerobic capacity, together with the rate of post-exercise recovery, can be one of the controlling tools in the training process of high-performance athletes in combat sports (Prokopczyk and Sokołowski, 2020). To date, the associations of stress coping style with the level of aerobic capacity and the level of post-exercise restitution and their changes during the training camp during the period of direct competitive preparation for championship competitions in wrestlers in the cadet age group have not been analyzed. The authors set out to analyze the results of the aerobic capacity test and the rate of post-exercise restitution in relation to the style of coping with stress in athletes in a training camp that was in the period of direct competitive preparation for the European Cadet Wrestling Championships. The research goal of the study was to determine the relationship between coping with stressful situations and the level of aerobic capacity and post-exercise restitution, as well as the changes occurring between these variables through the period of the training camp preceding the international championship competition.

The authors posed two research hypotheses:

*H1*: The athletes will maintain or improve the results obtained in the performance test and the post-workout restitution coefficient during the immediate preparation period for the European Championships.

*H2*: The style of coping with stressful situations significantly affects changes in aerobic capacity and post-workout restitution coefficient during the immediate preparation period for the European Championships.

The presented research will indicate the impact of psychological preparation on the physiological capabilities of young wrestlers during the period of immediate preparation for championship competitions. The research undertaken will provide important advice for coaches, sports psychologists, and people working with young athletes preparing for major competitions. Taking into account the period in the training process, the level of preparation should be at a constant or increasing level (due to the upcoming main competitions). Bearing in mind that the athletes are in the adolescence period and before major competitions, the authors hypothesized that their results may depend on their style of coping with stressful situations.

## 2 Participants

The study included a group of 16 men on the Polish National Cadet Wrestling Team in classical-style wrestling with an average age of 16.56 years (SD = 0.54). All those tested were called up to the national team training camp and were in the period of immediate preparation for the European Cadet Men’s Wrestling Championships.

## 3 Methods

Coping with stressful situations was assessed using the Coping Inventory for Stressful Situations (CISS) questionnaire by Endler and Parker (1990). The questionnaire consists of questions about behavior in stressful situations and allows us to determine the respondent’s tendency to use particular coping styles with stress (task—SST; emotional—SSE; avoidant—SSA). In addition, this questionnaire details two subcategories of avoidant style—engaging in substitute activities (ESA) and looking for social contacts (LSC). While filling out the questionnaire, in individual questions, the respondent determines how much he engages in given activities when he is in a stressful situation. Each question is answered using a 5-point scale (from 1 to 5). Individual ratings mean: 1—never; 2—very rarely; 3—sometimes; 4—often; 5—very often. The study used the CISS questionnaire adapted by Strelau et al. (2007) and Strelau and Jaworowska (2020) for use in Polish settings.

Aerobic capacity was explored using the Maximal Multistage 20-m Shuttle Run Test (the “Beep-Test”). This test involves running a designated 20-m distance marked by lines between sound signals (“beep”) at increasing speed and frequency at the next level. The examined must cross the designated line before the next signal (“beep”), otherwise he receives a warning. Receiving a second warning means the end of the test (Léger and Lambert, 1982). To calculate the estimated aerobic capacity, a modified formula was used for subjects aged 6–18: VO_2_ max [mL/kg/min] = −31.025 + 3.328 × 1–3.248 × 2 + 0.1536X1X2, where: X1 – maximum aerobic speed of the last level of “Beep-Test” [km/h]; X2 – age (as a rounded down integer) (Léger et al., 1988).

To calculate the level of post-workout restitution, Klonowicz coefficient of restitution calculations were performed at 3 min (COR 3′) and 5 min (COR 5′) after the end of the aerobic capacity test, according to the formulas: COR3′ = (Hr2–Hr3)/(Hr2–Hr1); COR5′ = (Hr2–Hr5)/(Hr2–Hr1), where: Hr1 – resting HR, Hr2 – HR after the “Beep-Test”, Hr3 – HR 3′ after the test “Beep-Test”, Hr5 – HR 5′ after the test “Beep-Test” (Kosendiak, 2013).

The subjects completed the CISS questionnaire once, at the beginning of the National Team Training Camp. The aerobic capacity test, along with the analysis of post-workout restitution, was carried out twice – at the beginning of the training camp (term I) and at the end of the training camp (term II). Tests of capacity were conducted without prior training so that the athletes rested while they performed them.

## 4 Statistical analysis

The following indicators were used to analyze the results: mean (*M*), minimum (Min), maximum (Max), standard deviation (SD or ±), and significance level and probability (*p*). The normality of the distribution was tested using the Shapiro–Wilk test. The analysis of the significance of changes between the study at terms I and II was applied to the *t*-test for dependent samples; in cases where the variable under study in at least one of the terms was not normally distributed, the Wilcoxon paired rank-order test was used. Cohen’s *d* coefficient was used to estimate the size of the effect. When the test value was less than 0.2, the result was considered insignificant; between 0.2 and 0.49, it was small; between 0.50 and 0.80, it was medium; and when it was greater than 0.80, it was considered as strong (Cohen, 1988). Pearson’s test and Spearman’s rank correlation coefficients were used to determine the strength of relationships between variables in the 2nd and 1st terms of the study. When the test value was less than 0.4, the result was considered low; between 0.4 and 0.69, it was considered medium; and 0.70 was considered strong (Akoglu, 2018).

## 5 Results

In the stress coping style test, the athletes scored an average of 60.1 (SD = 6.25) points in the task style, 41.9 points (SD = 8.19) in the emotional style, and 42.5 points (SD = 9.53) in the avoidant style. In the subcategories of avoidant style, they scored an average of 18.4 (SD = 5.15) points in the style, of engaging in substitute activities and 17.3 (SD = 3.59) points in the style of looking for social contacts. The tested men of the Polish National Cadet Wrestling Team between the 1st and 2nd terms of test terms showed a significant, large (Cohen’s *d* size = 2.80) increase in the level of aerobic capacity (VO_2_ max [mL/kg/min]) by an average of 7.7 mL/kg/min, a large (Cohen’s *d* size = 0.88) decrease in resting HR by an average of 8.1 bpm, a medium (Cohen’s *d* size = 0.59) decrease in HR 1 min after the test by an average of 13 bpm, and a medium (Cohen’s *d* size = 0.68) decrease in HR 5 min after the test by an average of 14.8 bpm. The other indicators tested showed no statistically significant (*p* ≥ 0.05) difference between the tested terms (Table 1).

**Table 1:** Descriptive characteristics of performance and restitution variables in the 1st and 2nd terms of the study in the Polish National Cadet Wrestling Team men (*n* = 16).

Variable (indicators “Beep = Test” and Klonowicz COR)	Term I	Term II	*t*	*p*	S–W test
	*M* ± SD				
Distance Beep-Test [m]	1413.3 ± 323.03	1637.5 ± 324.29	0.09^a^	0.926	0.829
Maximum aerobic speed of the last level of Beep-Test [km/h]	12.7 ± 0.68	12.7 ± 0.70	−0.62^a^	0.544	0.506
Beep-Test – VO_2_ max [mL/kg/min]	51.0 ± 4.06	58.7 ± 6.19	−7.70^a^	**<0.001**	0.554
Resting HR [bpm]	71.6 ± 8.20	63.5 ± 6.67	3.14^b^	**0.002**	0.066
HR after the test [bpm]	156.8 ± 29.69	161.8 ± 36.19	−0.69^a^	0.503	0.444
HR 1′ after the test [bpm]	131.8 ± 19.01	118.8 ± 20.43	2.16^b^	**0.031**	**0.011**
HR 3′ after the test [bpm]	108.8 ± 13.48	104.3 ± 10.38	1.86^a^	0.083	**0.006**
HR 5′ after the test [bpm]	101.3 ± 16.44	86.5 ± 11.11	3.15^a^	**0.007**	**0.039**
Klonowicz COR 3′ [%]	55.6 ± 19.06	53.4 ± 16.40	0.42^a^	0.680	0.469
Klonowicz COR 5′ [%]	67.3 ± 22.21	75.4 ± 12.73	1.65^b^	0.100	**0.009**

a: *t*-test for dependent samples; b: Wilcoxon paired t-test; values *p* < 0.05 are in bold.

The study at term I showed a positive medium significant correlation between SSE and HR 1′ after the test (*r* = 0.55; *p* < 0.05) and a negative medium significant correlation between SSA – LSC and HR 5′ after the test (*r* = −0.64; *p* < 0.01; Table 2).

**Table 2:** Correlations between aerobic capacity and post-workout restitution variables and stress coping style in the 1st and 2nd terms of the Polish National Team study in cadet wrestling men (*n* = 16).

Variable (indicators “Beep-Test” and Klonowicz COR)	SST		SSE		SSA		SSA – ESA		SSA – LSC	
	Term I	Term II	Term I	Term II	Term I	Term II	Term I	Term II	Term I	Term II
Distance in Beep-Test [m]^a^	0.08	−0.12	0.35	0.22	0.07	−0.12	0.06	−0.21	−0.11	−0.18
Maximum aerobic speed of the last level of Beep-Test [km/h]^a^	0.09	−0.08	0.35	0.17	0.14	−0.23	0.13	−0.30	−0.09	−0.26
VO_2_ max [mL/kg/min]^a^	0.10	0.27	0.34	0.17	0.13	−0.10	0.12	−0.09	−0.10	−0.35
Resting HR [bpm]^a^	0.00	0.06	−0.10	−0.20	0.10	0.20	0.09	0.09	0.10	0.09
HR after the test [bpm]^a^	−0.38	−0.34	−0.07	−0.14	−0.42	−0.34	−0.36	−0.29	−0.39	−0.39
HR 1′ after the test [bpm]^b^	−0.09	−0.29	**0.55**^**I**^	0.46	0.16	0.22	0.14	0.03	0.22	0.35
HR 3′ after the test [bpm]^b^	−0.26	−0.21	−0.11	0.46	−0.27	0.28	−0.33	0.08	−0.40	0.30
HR 5′ after the test [bpm]^b^	−0.31	0.03	−0.18	−0.37	−0.39	−0.05	−0.34	−0.20	**−0.64**^**II**^	0.24
Klonowicz COR 3′ [%]^a^	0.12	−0.24	−0.02	−0.41	−0.24	−0.43	−0.11	−0.30	−0.21	−0.49
Klonowicz COR 5′ [%]^b^	0.14	−0.16	0.11	0.44	−0.07	−0.04	−0.07	0.08	0.14	−0.37

a: Pearson test; b: Spearman’s rank correlations; term I = *p* < 0.05; term II = *p* < 0.01; values *p* < 0.05 are in bold.

Between the 2nd and 1st terms, there was a negative medium significant correlation between SSA and maximum aerobic speed at the last level of “Beep-Test” (*r* = −0.64; *p* < 0.008), a negative medium significant correlation between SSA–ESA and distance in Beep-Test (*r* = −0.55; *p* < 0.029), a negative strong significant correlation between SSA–ESA and maximum aerobic speed of the last level of “Beep-Test” (*r* = −0.72; *p* < 0.002), a positive medium significant correlation between SSA–LSC and HR 3′ after the test (*r* = 0.60; *p* < 0.015), and a positive medium significant correlation between SSA–LSC and HR 5′ after the test (*r* = 0.57; *p* < 0.020; Table 3).

**Table 3:** Results of analysis of changes in the relationship between performance variables and stress coping style between Term II and Term I in the Polish National Team group in cadet wrestling men (*n* = 16).

Variable (indicators “Beep = Test” and Klonowicz’s COR)	SST		SSE		SSA		SSA – ESA		SSA – LSC	
	*r*	*p*	*r*	*p*	*r*	*p*	*r*	*p*	*r*	*p*
Distance in Beep-Test [m]^a^	−0.42	0.109	−0.25	0.357	−0.39	0.138	**−0.55**	**0.029**	−0.13	0.634
Maximum aerobic speed of the last level of Beep-Test [km/h]^b^	−0.20	0.457	−0.41	0.112	**−0.64**	**0.008**	**−0.72**	**0.002**	−0.27	0.304
VO_2_ max based on Beep-Test [mL/kg/min]^a^	0.31	0.239	−0.09	0.754	−0.29	0.283	−0.27	0.322	−0.44	0.088
Resting HR [bpm]^a^	0.08	0.762	0.23	0.388	0.10	0.722	0.02	0.931	0.02	0.934
HR after the test [bpm]^b^	0.02	0.950	−0.17	0.518	0.05	0.859	−0.02	0.937	0.17	0.542
HR 1′ after the test [bpm]^a^	−0.09	0.744	−0.06	0.818	0.10	0.711	−0.04	0.871	0.30	0.265
HR 3′ after the test [bpm]^a^	0.23	0.401	0.42	0.105	0.50	0.051	0.35	0.181	**0.60**	**0.015**
HR 5′ after the test [bpm]^a^	0.29	0.274	−0.21	0.433	0.16	0.549	0.06	0.814	**0.57**	**0.020**
Klonowicz COR 3′ [%]^b^	−0.20	0.451	−0.49	0.053	−0.27	0.321	−0.25	0.348	−0.28	0.289
Klonowicz COR 5′ [%]^a^	−0.29	0.283	0.22	0.408	0.02	0.929	0.04	0.882	−0.31	0.250

a: Pearson test; b: Spearman’s rank correlations; values *p* < 0.05 are in bold.

## 6 Discussion

Both research hypotheses were partially confirmed. The results of the study show that there was a significant increase in VO_2_ max [mL/kg/min] and a decrease through the grouping period: resting HR, HR 1′ after the test, HR 5′ after the test in the athletes. The level of COR 3′ decreased and COR 5′ increased, but both at statistically insignificant levels. Stress coping style was also shown to be significantly associated with indicators of the performance test and HR 1′ after the test. No significant associations were noted with post-workout restitution indicators. The results of the study show that athletes with a higher level of emotional stress coping style obtained a higher number of heart contractions 1 min after the test on the first test date. Athletes with an evasive stress coping style of seeking social contact obtained a lower number of heart contractions 5 min after the test on the 2nd test date, but this style had a significant effect on increasing heart rate 3 min after the test and 5 min after the test compared to the 1st test date. Wrestlers with a higher evasive style of coping with stress had a significantly lower maximum aerobic speed of the last level score in the 2nd term compared to the 1st term of the study. In addition, subjects with an evasive stress-coping style of engaging in substitute activities scored significantly lower distance and maximum aerobic speed of the last level in the 2nd term of the study. Previous research has shown that the only stress coping style suitable for competition in competitive sports is a task-based style (Secades et al., 2016). Younger wrestlers are more likely to react emotionally to stressful stimuli and seek interpersonal connections, which significantly reduces performance when competing in professional sports (Tomczak et al., 2013; Namazov et al., 2019). It has been proven that senior wrestlers deal with stress differently than younger athletes competing at the professional level (Rutkowska et al., 2020). This may be influenced by adolescence and the transition to sports at the senior level. During this period, psychosocial stress is among the biggest concerns of young athletes (Lundqvist et al., 2023). Therefore, it is reasonable to provide psychological preparation as early as possible, carried out in a methodical manner, giving athletes the ability to solve stressful situations and recover from them as quickly as possible. Importantly, other studies have shown that older athletes competing in the international elite experience a decline in mental toughness with age compared to younger athletes competing at the same level (Korobeynikov et al., 2022b). This indicates that there is a constant need to monitor the mental toughness of athletes competing in professional sports, even after they have achieved the highest results at major international competitions. This will allow for the identification of the moment of reduction in the level of psychophysiological capabilities of a particular wrestler. The results of the conducted research and other studies indicate that competing at the highest level in wrestling requires parallel sports preparation that includes fitness, technical, tactical, and mental training, which should be properly conducted from a young age, matching the age, level, and needs of the training period the athlete is in Stenling et al. (2015), Sabato et al. (2016), Piepiora et al. (2021b), and Piepiora et al. (2023). Research results indicate that the last training period for young wrestlers preparing for championship competitions should include psychological training. However, it should be taken into account that mental training is an integral part of competitive combat sports, significantly influencing the results obtained at the decisive moment, regardless of the age group in which one competes (Andreato et al., 2022). That indicates that proper psychological preparation and the ability to control one’s functioning at key moments should be continuously developed during the wrestlers’ training process. That indicates that proper psychological preparation and the ability to appropriately and effectively cope with stressful situations should be continuously developed during the wrestlers’ training process. This also indicates that continuous research should be conducted, taking into account the effectiveness of psychological preparation programs in professional sports in individual age groups and the implications of the most effective methods of psychological preparation for professional.

The presented research was characterized by limitations affecting the size of the sample, as it was conducted on a small, selected number of athletes attending a camp preparing for the European Men’s Cadet Wrestling Championship. Taking into account the period of adolescence, selected groups, and various periods occurring in the training cycle, it would be reasonable to conduct multi-year research analyzing the functioning of young wrestlers in different periods of the training cycle. Covering the research of competitive athletes of all age groups and a larger group of athletes than those directly preparing for championship competitions would make it possible to compare successful players competing in the senior group with adolescent athletes. Moreover, in the case of conducting comparative longitudinal studies, it will be possible to demonstrate young athletes who have the psychological predispositions to compete at the championship level in senior sports. At the same time, given the lack of similar studies and the correlations that were shown in the research, in the opinion of the authors, it would be valuable for a more detailed understanding of the studies to analyze the psychophysiological changes of the entire cycle of preparation for championship competitions along with the sports results achieved at them.

## 7 Conclusion

Significant correlations were found between the style of coping with stress and scores in the performance test and the number of heart contractions in athletes preparing for the European Cadet Men’s Wrestling Championships. The demonstrated significant changes between the 1st and 2nd terms of the study indicate that it is necessary to introduce psychological preparation into the training program of the national team, along with regular evaluation of its effectiveness.

## Data availability statement

The raw data supporting the conclusions of this article will be made available by the authors without undue reservation.

## Ethics statement

The researchers have obtained the consent of the Bioethics Committee, at the Medical University of Poznań, issued on 14 April 2022 with the number 294/22. The participants’ legal guardian/next of kin provided written informed consent to participate in this study. The patients/participants provided their written informed consent to participate in this study. The studies were conducted in accordance with the local legislation and institutional requirements. The participants provided their written informed consent to participate in this study.

## Author contributions

PJ: Conceptualization, Data curation, Formal analysis, Funding acquisition, Investigation, Methodology, Project administration, Resources, Software, Supervision, Validation, Visualization, Writing – original draft, Writing – review & editing. MS: Conceptualization, Funding acquisition, Methodology, Project administration, Supervision, Writing – original draft, Writing – review & editing. AP: Conceptualization, Data curation, Formal analysis, Funding acquisition, Investigation, Methodology, Project administration, Resources, Software, Supervision, Validation, Visualization, Writing – original draft, Writing – review & editing.

## References

[ref1] Akoglu H. (2018). User’s guide to correlation coefficients. Turk. J. Emerg. Med. 18, 91–93. doi: 10.1016/j.tjem.2018.08.001, PMID: 30191186 PMC6107969

[ref9001] Andreato L. V. dos Santos M. G. Andrade A. (2022). What do we know about the effects of mental training applied to combat sports? A systematic review. Psychol. Sport Exerc 63, 102267. doi: 10.1016/j.psychsport.2022.102267

[ref2] Atiya D. Berliana Komarudin. (2022). The relationship of psychological skill with stress recovery on wrestling athletes in Indonesia. Gladi 13, 307–315. doi: 10.21009/GJIK.133.06

[ref3] Clarke D. H. Vaccaro P. Andresen N. M. (2013). Physiological alterations in 7- to 9-year-old boys following a season of competitive wrestling. Res. Q. Exerc. Sport 55, 318–322. doi: 10.1080/02701367.1984.10608409

[ref4] Cohen J. (1988). Statistical power analysis for the behavioral sciences. 2nd Edn. Hillsdale: Erlbaum Associates.

[ref5] Demirkan E. (2015). Physical and physiological characteristics in adolescent wrestlers. Monten. J. Sports Sci. Med. 4, 13–18.

[ref6] Endler N. S. Parker J. D. A. (1990). Multidimensional assessment of coping: a critical evaluation. J. Pers. Soc. Psychol. 58, 844–854. doi: 10.1037/0022-3514.58.5.844, PMID: 2348372

[ref7] Korobeynikov G. Baić M. Potop V. Korobeinikova L. Raab M. Starčević N.. (2022a). Comparative analysis of psychophysiological states among Croatian and Ukrainian wrestling. J. Phys. Edu. Sport. 22, 1832–1838. doi: 10.7752/jpes.2022.08230

[ref8] Korobeynikov G. Korobeinikova L. H. Korobeinikova I. (2022b). “Stress management in wrestling” in Influence of physical culture and sport on the formation of an individual healthy lifestyle (Izdevniecība: “Baltija Publishing), 106–122.

[ref9] Kosendiak J. (2013). Projektowanie systemów treningowych. Studia i monografie. Wrocław: Akademia Wychowania Fizycznego [In Polish].

[ref10] Léger L. A. Lambert J. (1982). A maximal multistage 20-m shuttle run test to predict VO_2_ max. Eur. J. Appl. Physiol. 49, 1–12. doi: 10.1007/BF00428958, PMID: 7201922

[ref11] Léger L. A. Mercier D. Gadoury C. Lambert J. (1988). The multistage 20 metre shuttle run test for aerobic fitness. J. Sports Sci. 6, 93–101. doi: 10.1080/02640418808729800, PMID: 3184250

[ref12] Lundqvist C. Schary D. P. Eklöf E. Zand Z. Jacobsson J. (2023). Elite lean athletes at sports high schools face multiple risks for mental health concerns and are in need of psychosocial support. PLoS One 18:e0284725. doi: 10.1371/journal.pone.0284725, PMID: 37083747 PMC10121048

[ref13] Marković M. R. Kasum G. Dopsaj M. Toskić L. Zarić I. (2018). Various competitive level wrestlers’ preparedness assessed by the application of the field test. Fizicka Kultura 72, 170–180. doi: 10.5937/fizkul1802170M

[ref14] Namazov K. A. Guzikova L. A. Namazov A. K. (2019). “Effect of wrestlers’ experience on the choice of coping strategies” in Professional culture of the specialist of the future. eds. Almazova N. I. Rubtsova A. V. Bylieva D. S., European Proceedings of Social and Behavioural Sciences, Future Academy, vol. 73, 234–243. doi: 10.15405/epsbs.2019.12.26

[ref15] Piepiora P. (2019). “Behaviors of professional athletes in terms of the big five model due to the type of contact of the sport discipline,” in Proceedings of the 14th international RAIS conference on social sciences and humanities, Princeton, USA: Research Association for Interdisciplinary Studies, 138–145.

[ref16] Piepiora P. (2021). Personality profile of individual sports champions. Brain Behav. 11:e02145. doi: 10.1002/brb3.2145, PMID: 33951345 PMC8213921

[ref17] Piepiora P. Kwiatkowski D. Bagińska J. Agouridas D. (2021a). Sports level and the personality of American football players in Poland. Int. J. Environ. Res. Public Health 18:13026. doi: 10.3390/ijerph182413026, PMID: 34948636 PMC8701363

[ref18] Piepiora P. Naczyńska A. (2023). Personality traits vs. sports classes of polish representatives in junior sports acrobatics. Sports 11:194. doi: 10.3390/sports11100194, PMID: 37888521 PMC10610551

[ref19] Piepiora P. Petecka A. (2020). Personality profile of women practising contact sports using the example of karate kyokushin competitors and handball players. Ido Mov. Cult. 20, 23–29. doi: 10.14589/ido.20.1.3

[ref20] Piepiora P. Piepiora Z. (2021). Personality determinants of success in men’s sports in the light of the big five. Int. J. Env. Res. Pub. Health. 18:6297. doi: 10.3390/ijerph18126297, PMID: 34200739 PMC8296103

[ref21] Piepiora P. Piepiora Z. Bagińska J. (2022). Personality and sport experience of 20–29-year-old Polish male professional athletes. Front. Psychol. 13:854804. doi: 10.3389/fpsyg.2022.854804, PMID: 35422742 PMC9004389

[ref22] Piepiora P. A. Piepiora Z. N. Stackeová D. Bagińska J. (2023). Physical culture from an interdisciplinary perspective. Front. Psychol. 14:1254027. doi: 10.3389/978-2-8325-3185-3 37560096 PMC10407795

[ref23] Piepiora P. Rauk-Kubacka A. Kubacki R. (2021b). Sport psychology in the physical culture sciences. A review. Pedagog. Psychol. Sport. 7, 61–75. doi: 10.12775/PPS.2021.07.01.003

[ref24] Piepiora P. Superson M. Witkowski K. (2017). Personality and the nutritional habits of athletes using the example of the polish national youth female wrestling team. Arch. Budo Sci. Martial Art Extreme Sport 13, 103–110.

[ref25] Piepiora P. Superson M. Witkowski K. Maslinski J. Migasiewicz J. (2018). Body composition characteristics vs a subjective assessment of body mass and eating habits by female wrestlers using the example of the polish national youth team. Arch. Budo Sci. Martial Art Extreme Sport 14, 87–91.

[ref26] Piepiora P. Witkowski K. (2020a). Personality profile of combat sports champions against neo-gladiators. Arch. Budo 16, 281–293.

[ref27] Piepiora P. Witkowski K. (2020b). Self-defence as a utilitarian factor in combat sports, modifying the personality of athletes at a champion level. Arch. Budo Sci. Martial Arts Extreme Sport. 16, 63–69.

[ref28] Prokopczyk A. Sokołowski M. (2020). Training experience and weekly training total time vs. aerobic capacity and level of effective restitution of male polish judo National Senior Team athletes during the preparation period for the Olympic games. Arch. Budo Sci. Martial Art Extreme Sport. 16, 63–69.

[ref29] Pryimakov O. Iermakov S. Eider J. Prysiazhniuk S. Skrypko A. Mazurok N. (2020b). Ratio and interconnections of functional fitness structure key components of elite combat athletes at the stage of maximum realization of individual capabilities. Phys. Educ. Stud. 24, 286–292. doi: 10.15561/20755279.2020.0505

[ref30] Pryimakov O. Iermakov S. Eider J. Prysiazhuniuk S. Mazurok N. (2020a). Physiological criteria of functional fitness and determinants of physical work capacity of highly skilled wrestlers. Phys. Edu. Stud. 24, 205–212. doi: 10.15561/20755279.2020.0403

[ref31] Rutkowska K. Gierczuk D. Buszta M. (2020). Selected psychological factors in elite Greco-Roman wrestlers at various levels of competition. J. Phys. Educ. Sport 20, 2277–2282. doi: 10.7752/jpes.2020.s3306

[ref32] Sabato T. M. Walch T. J. Caine D. J. (2016). The elite young athlete: strategies to ensure physical and emotional health. J. Sports Med. 7, 99–113. doi: 10.2147/OAJSM.S96821, PMID: 27621677 PMC5012846

[ref33] Sabirov O. (2022). Features of factors that influence high sports achievements in wrestling. Sci. J. Natl. Pedagogical Dragomanov Univ. 11, 21–23. doi: 10.31392/NPU-nc.series15.2022.11(157).05

[ref34] Sawczyn S. Jagiełło W. Fetisov V. I. Mishchenko V. (2015). Dependence of work capacity recovery after strenuous training sessions upon individual predisposition of skilled wrestlers to work under different energy modes. Arch. Budo 11, 197–207.

[ref35] Secades X. G. Molinero O. Salguero A. Ruiz R. De la Vega R. Márquez S. (2016). Relationship between resilience and coping strategies in competitive sport. Percept. Mot. Skills 122, 336–349. doi: 10.1177/0031512516631056, PMID: 27420325

[ref36] Stenling A. Lindwall M. Hassmén P. (2015). Changes in perceived autonomy support, need satisfaction, motivation, and well-being in young elite athletes. Sport Exerc. Perform. 4, 50–61. doi: 10.1037/spy0000027

[ref37] Strelau J. Jaworowska A. (2020). CISS. Kwestionariusz Radzenia Sobie w Sytuacjach Stresowych. Wydanie Czwarte. Warszawa: Pracownia Testów Psychologicznych [In Polish].

[ref9002] Strelau J. Jaworowska A. Wrześniewski K. Szczepaniak P. (2007). Kwestionariusz Radzenia Sobie w Sytuacjach Stresowych, CISS. Warszawa: Pracownia Testów Psychologicznych. [in Polish].

[ref38] Tomczak M. Bręczewski G. Sokołowski M. Kaiser A. Czerniak U. (2013). Personality traits and stress coping styles in the Polish National Cadet Wrestling Team. Arch. Budo 9, 161–168.

[ref39] United World Wrestling (2023). International wrestling rules, Greco-Roman wrestling, freestyle wrestling, women’s wrestling. United World Wrestling; Location of the publication is Corsier-sur-Vevey.

[ref40] Witkowski K. Piepiora P. Migasiewicz J. Maśliński J. Salachna A. (2018). Physical fitness, developmental age and somatic development of youth Greco-Roman wrestlers and school youth aged 13-14 years. Arch. Budo Sci. Martial Art Extreme Sport 14, 63–74.

